# Physiological load estimation in athletes using ECG-derived features and gradient-boosted modeling

**DOI:** 10.3389/fpubh.2026.1843963

**Published:** 2026-05-22

**Authors:** Mei Li, Jichao Xu

**Affiliations:** 1 College of Physical and Health Education, Taizhou College of Nanjing Normal University, Taizhou, Jiangsu, China; 2 College of Sports Science, Hefei Normal University, Hefei, Anhui, China

**Keywords:** breathing rate, electrocardiography (ECG), heart rate variability (HRV), machine learning, physiological load, reproducible sports analytics, SHAP, SportDB 2.0

## Abstract

**Introduction:**

Measuring internal load is crucial for athlete training management, but many athlete monitoring tools use proprietary pipelines that lack transparency and reproducibility.

**Methods:**

This study presents a retrospective ECG/HRV-based machine learning framework for calculating session-level Training Impulse (TRIMP) using the publicly available SportDB 2.0 dataset. The reference target variable was calculated using the time spent on the session and heart-rate response, which is known as TRIMP. Supervised learning models were constructed using descriptors from the ECG, such as heart rate and heart rate variability features. TRIMP and variables directly derived from TRIMP were excluded from the predictor set to reduce circularity and improve methodological consistency. SHAP was used to evaluate the interpretability of models.

**Results:**

XGBoost showed the strongest overall cross-validation performance among the tested models. The results indicate that ECG-derived descriptors, particularly heart-rate-variability features, can reconstruct a conventional heart-rate-based internal-load reference with strong predictive performance.

**Discussion:**

The proposed framework is viewed as a clear and repeatable approach to obtain a benchmark for TRIMPs at the session level and not as a model to predict an independent physiological outcome. This study offers a free and interpretable pipeline for session-level TRIMP estimation, enabling reproducible sports analytics.

## 1 Introduction

Evidence-based training has become a new orientation within the culture of elite sports over the last few decades, and in this case, data from data-driven technologies are relied upon to influence the coaching process, athlete management practices, and injury prevention plans. The measurement and surveillance of inner physiological responses in an athlete, without dependence on performance outcomes, provides a clearer view of the consequences of cumulative training stress and recovery. This has been enabled by converged sensory technology, computational analytics, and sport science methods. With increasing pressure of loads and competition, an increased pace of components, and a shorter recovery period, coaches and sports scientists are striving to ensure that the factor of load and recovery is optimized (1). Any failure to establish such a balance can result in performance declines, an increased risk of injury, or overtraining syndromes (2). In that regard, the constant monitoring of physiological parameters throughout the training-competition-recovery cycle creates an opportunity to control the athlete’s state in a more proactive, rather than reactive, way. Continuous monitoring enables detection of maladaptation before injury or performance declines, rather than relying on decreased performance to adjust training prescriptions or make informed load-management decisions. This study is a retrospective secondary analysis of SportDB 2.0. Each session was treated as one observational unit, and the primary objective was session-level TRIMP estimation from ECG-derived features. Elite sport is a field where a slight margin between victory and defeat can exist. The athletes have to train at very demanding rates on a regular basis; they are obliged to juggle travel and competition schedules; and they must be physiologically fit throughout the long seasons. The ability to quantify recovery and load is critical in this case. Traditional surveillance mechanisms, such as periodic laboratory tests or regular health check-ups, cannot achieve high temporal resolution and therefore may not detect acute physiological changes that lead to maladaptation or injury. The workload levels measured by wearable devices were also associated with injuries in team sports, highlighting the potential for ongoing monitoring (3). Continuous monitoring of elite physiology has several benefits. The latter is achieved by displaying real-time or near-real-time feedback on internal load (i.e., heart rate responses, heart rate variability, respiratory strain) so that coaches can modify training intensity or volume in real time. Second, it will allow tracking of recovery condition and preparedness, which will help make more accurate decisions about whether the athlete is ready to run at high intensity or match load. Third, it helps detect fatigue, autonomic dysregulation, or overtraining before symptoms appear. In fact, the latest reviews also confirm that predicting injury risk and guaranteeing optimal performance are achieved by combining control of internal and external load parameters with constant tracking (4, 5). Based on these advantages, the case that underlies the use of a wearable sensor-based continuous monitoring system by elite athletes is solid.

Wearable sensor technologies have reached the point where physiological and biophysiological signals can be measured in the field with a bit of obtrusion. Seckin et al. present a generalization of wearable technologies in sport in which current sensor systems merge physiological (e.g., ECG, EMG, respiration), motion/positional (e.g., accelerometers, gyroscopes, GPS), and environmental (e.g., temperature, humidity) data streams (5). One of the most significant reviews on the topic of wearables in sports medicine is that by Seshadri et al., which emphasizes that wearables now move beyond the laboratory setting and into 24/7 monitoring of athletes, not only on the field but also during the training process (6). The machines can be conveniently installed in athletes’ training settings, enabling tracking of longitudinal data during training blocks and competition periods. As an example, the calculation of heart rate, HRV, respiration, sweat content, and motion indicators is performed in wearable devices, such as clothes or patches, with high temporal resolution today (7). Although validating devices is challenging, research on data processing and ensuring inter-device compatibility is challenging as well, the industry is rapidly transitioning to more athlete-centered applications (8). In concise terms, sport wearable sensor systems provide technology for sustained physiological monitoring in elite sport, enabling the current study to focus on load and recovery dynamics.

Athlete monitoring is the solution for the correct conceptualization and measurement of load and recovery. The summation of the stress induced by training and competition on the athlete is termed training load, and the mechanism by which the body recovers to a normal condition, or even a higher level of preparedness, is called recovery (9). Load may be further subdivided into external load (e.g., distance, accelerations, number of sprints) and internal load (e.g., physiological and psychological responses of an athlete to the external load, such as heart rate, RPE, metabolic indicators) (10). The recovery measurements are intended to reflect readiness to train, capacity to sustain the load, and homeostatic restoration. Physiological variables in elite sports encompass heart rate (HR), heart rate variability (HRV; e.g., RMSSD, SDNN), resting heart rate, sleep quality/time, or biochemical parameters, such as creatine kinase or lactate (11). HRV is now gaining popularity as a non-invasive indicator of autonomic balance and the well-being of athletes; systematic reviews show that daily HRV monitoring during elite team sports training is useful for assessing training adaptations and predicting readiness status (12). Furthermore, the acute: chronic workload ratio (), which compares recent load to longer-duration load, has been used as an indicator to inform load management and injury risk (13). However, it has limited shortcomings: standardization of measurement procedures, individual variations, and system authenticity of the device are critical problems (14). The interaction between load and recovery in the elite athlete is dynamic: excessive load relative to recovery will lead to maladaptation, poor performance, or injury, whereas optimal loading with proper recovery will lead to adaptation and performance improvement. With wearables, real-time observation of this interaction is continuous, enabling better training, prescription, and management.

Since the outcome of interest in the current research, Training Impulse (TRIMP), is determined by the heart-rate response and session duration, and various candidate predictors are also derived from ECG-based heart-rate dynamics, the current modeling task has inherent target-related overlap. In that regard, this research cannot be construed as the prophecy of a completely autonomous physiological construct of a unique sensing modality. Instead, it builds a clear, understandable model to estimate a popular heart-rate-based internal-load reference from ECG-derived descriptors in a publicly accessible dataset. The rest of this paper is structured as follows. Section 2 surveys related research in wearable sensing and athlete monitoring. Section 3 outlines materials, methods, and the analysis framework. Section 4 presents the results of the experiment and model assessment, and these are discussed in Section 5. The conclusions are presented in Section 6.

## 2 Related work

Wearable sensing has moved beyond intermittent, laboratory-constrained testing to continuous, field-based monitoring that meets the full range of training and competition requirements. Modern systems are incorporating photoplethysmography (PPG), single-lead ECG, inertial measurement units (IMUs), global navigation satellite systems (GNSS), and skin/ambient thermistors to measure internal events and external work at high temporal resolution (15, 16). In both athletic and clinical-sport cohorts, validation studies typically indicate that errors in heart-rate tracking are acceptable under free-living conditions or during exercise. Nevertheless, at high intensities or when the motion artefact is present, accuracy may decrease (17, 18). The extrinsic skill-specific outputs (e.g., kicking, jumping) are now measured in Platform (19–21) with increasing validity across ecological environments (19–21). The progressions allow for longitudinal profiling of athletes, linking physiological strain to performance and health, and maintaining ecological validity through actual sessions and matches in the field (15, 16).

The load is usually divided into an external load (physiological/locomotor stimulus) and an internal load (psychophysiological response of an individual) in a consistent causal pattern (22, 23). HR and HRV are key central internal-load metrics; recent systematic reviews in elite team sports favor the use of HRV to monitor autonomic balance and readiness, as measured on standardized protocols (12, 24). The acute: chronic workload ratio (ACWR) has commonly been employed in load-to-injury studies, though this has yielded mixed results: some studies have found relationships with injury risk, whilst others have found it to be methodologically flawed and misused (24–27). In addition to individual measures, multi-indicator surveillance, which integrates HR/HRV, session RPE, sleep, and skill-specific external load, seems to be better for decision-making during busy schedules and campaigns requiring travel (15, 28).

Signal-processing pipelines are used to address motion artefacts (PPG/ECG), drift (IMUs), and IMU synchronization across multiplexed streams. Recent studies show credible HR extraction and beat-to-beat results from wrist wearables across different workloads when devices are rigorously tested against criterion references, and analyses account for movement noise (17, 18, 29). IMU-based analytics quantify jump count/height and limb-specific outputs with good agreement to optical motion capture in sport-specific contexts, facilitating granular external-load profiling on and off rigid surfaces (20, 21). At the systems level, coupling internal (HR/HRV) and biomechanical (IMU/GNSS) signals enables estimation of physiological strain, cumulative fatigue, and recovery trajectories rather than relying on any single surrogate (15, 16). Emerging studies extend beyond training to competition and to guided interventions, e.g., HRV-informed programming, showing promising performance and readiness benefits in randomized designs (30).

In the context of stochastic physiological response and inter-individual variation, fuzzy logic and hybrid soft-computing frameworks provide interpretable decision support in times of uncertainty (e.g., linguistic rules of high load and inadequate recovery) (31). Sports Fuzzy Inference. In sports, wearable sensors have been used to evaluate players and prescribe sessions based on fuzzy inferences of wearable signals, rulesets modeled expert knowledge, and noisy inputs are tolerated (31–33). Simultaneously, machine and deep learning methods include: temporal models, representation learning of raw sensor time series, multimodal fusion, and are finding applications in injury-risk screening, readiness classification, and performance prediction (34, 35). Critically, the existing best practice focuses on the validation of models using gold-standard references, open reporting (feature sets, hyperparameters), and calibration to use in elite cohorts (15).

There are still four gaps despite such rapid progress. (i) Ecological continuity: most experiments record discrete sessions or short blocks instead of end-to-end cycles comprising training, competition, and recovery, which restricts any inferences about cumulative dose and adaptation (15, 28). (ii) Validation breadth: the accuracy of devices usually decreases when there is high-intensity play, when the contact is made, or when there is multi-directional movement; cross-sport validation is not even (17–20). (iii) Causal interpretation: surrogate measures (e.g., ACWR) run the risk of simplifying dose–response relationships; better causal designs and models taking the individual case would be desirable (22, 23, 26, 27). (iv) Translational analytics: although fuzzy and ML techniques have potential, interpretable, real-time, and athlete-specific decision systems, bench-tested with strong results, are a nascent technology (31, 34, 35). The current paper fills these gaps by introducing continuous, multi-sensor internal-external monitoring during training and competition, criterion-outcome validation, and interpretable analytics to guide practitioners’ decisions. Table 1 provides an overview of the major studies on wearable sensor-based monitoring in athletes.

**Table 1:** Summary of key studies on wearable sensor–based monitoring in athletes.

Ref.	Population/sport	Sensors & signals	Primary metrics	Design & setting	Key findings/contribution	Noted limitations
(17)	Youth/clinical sport participants	Wrist PPG HR trackers	HR, bias/error vs. criterion	Validation, exercise tests	Minimal bias; reliable HR in monitored tasks	Accuracy degrades at high intensity/motion
(18)	Healthy adults (exercise)	Smartwatch & chest patch	HR, RR intervals	Validation vs. ECG	Acceptable accuracy in most conditions	Device/condition dependence; motion artefact
(19)	Football kicking	Foot-mounted IMU	Kicking output, counts	Concurrent validity vs. video	Good agreement for event detection/quantification	Sport-specific generalizability
(20)	Beach volleyball	Wearable IMU (jump)	Jump detection/height	Field validation on sand	Valid jump metrics in non-rigid surfaces	Context-specific calibration needed
(21)	Functional lifting tasks	Multi-segment IMU	Joint kinematics agreement	Validation vs. optical capture	High sagittal-plane agreement	Non-gait motions are less explored
(30)	Endurance athletes	HR/HRV (vmHRV), RHR	HRV-guided training response	RCT, field	HRV-guided programming improved outcomes	Sport/sample size constraints
(26)	Team sports	External load logs	ACWR vs. injury association	Systematic review	Mixed evidence; cautions on ACWR use	Heterogeneity, risk of bias
(24)	Soccer	HRV indices	HRV vs. overtraining symptoms	Systematic review	Associations suggested; protocol importance	Limited high-quality longitudinal data
(27)	Mixed sports	IMU workloads	ACWR and injury risk	Observational analysis	Contextualized ACWR with IMU data	Causality/threshold issues

## 3 Methodology

### 3.1 Study design and experimental framework

This paper is a retrospective secondary analysis of the publicly available SportDB 2.0 dataset reported by Burattini et al. (36). All physiological data analyzed in this work were previously stored and publicly accessible as part of the original dataset. The primary modeling objective was session-level TRIMP estimation. No clinical outcome or recovery-status prediction was modeled.

In the current reanalysis, every session that was taken was considered an observational unit. The analysis aimed to estimate the session-based internal physiological load (TRIMP) using ECG-derived measures and related heart rate variability measures. The study workflow was thus as follows: offline signal preprocessing, ECG-derived series feature extraction, model development, cross-validation, and *post hoc* interpretability analysis.

Since TRIMP is calculated using heart-rate response and session duration, and several retained predictors are also derived from heart-rate dynamics measured by ECG, there is conceptual overlap between the predictors and the target in the modeling task. Based on this, the current framework can be considered a clear and reproducible reconstruction of a traditional internal-load reference, rather than a forecast of an independent physiological endpoint.

**Figure 1:**
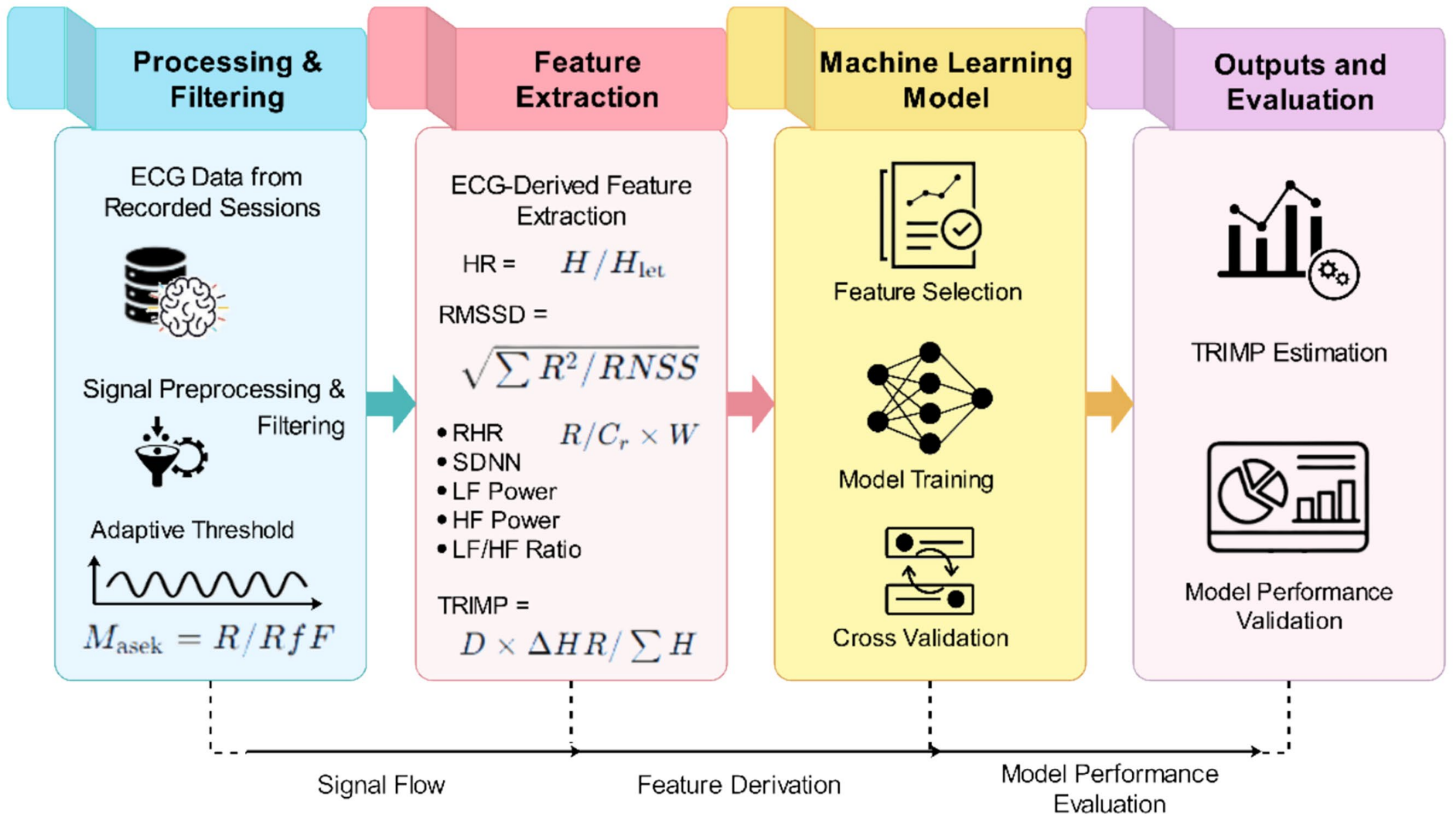
Retrospective ECG-based analysis workflow for session-level TRIMP estimation using SportDB 2.0.

The proposed approach, outlined in Figure 1, integrates wearable sensor data recorded during the baseline, training, competition, and recovery phases into the study workflow. It also depicts the subsequent data-processing steps: synchronization, data preprocessing, and modeling of physiological load and recovery responses.

The methodological aspect worth considering is that the target variable, TRIMP, is, in turn, derived from heart-rate response and session duration, and some of the retained predictors are derived from ECG-derived heart-rate dynamics. Thus, even though TRIMP and ACWR are not considered as explicit predictors, there is still some conceptual overlap between predictors and the target in the analysis. The current effort can be seen as the reconstruction or estimation of a reproducible internal-load reference, rather than predicting an independent physiological endpoint. This weakness is taken into account when interpreting the model’s performance and the study’s contribution.

### 3.2 Data sources and datasets

The analyses in this manuscript use SportDB 2.0 (36), a public dataset providing ECG recordings and cardiorespiratory measures collected during sport- and exercise-related sessions. We restrict our feature set to variables available from the ECG and its associated derived series, as well as session metadata such as duration. No additional proprietary sensor streams are assumed. All preprocessing and feature definitions are described to support reproducibility. This study is a retrospective analysis of the publicly available SportDB 2.0 dataset. The authors did not conduct any new data collection, baseline calibration, or longitudinal monitoring protocol. All analyses were performed using previously recorded physiological and activity data contained in the dataset.

### 3.3 Signal specifications and feature computation

Single-lead ECG recordings were used to derive heart rate (HR) and heart-rate variability (HRV). R-peak detection followed a Pan–Tompkins–style approach with adaptive thresholding to improve robustness to noise. Signal preprocessing, including artifact removal and zero-phase filtering, was performed offline to ensure signal quality prior to feature extraction and model training. Standard HRV metrics were computed in the time domain (e.g., RMSSD, SDNN) and, where applicable, in the frequency domain using fast Fourier transform–based spectral estimation The session-level TRIMP formulation used in this study is presented in Equation 1.


$$\text{TRIMP}=\sum_{i=1}^{n}D_{i}\cdot \mathrm{\Delta H}R_{i}\cdot e^{b\cdot \mathrm{\Delta H}R_{i}}$$ (1)

Where:

-
$D_{i}$ = duration of interval i
-
$\mathrm{\Delta H}R_{i}$ = normalized heart rate reserve
-
b = sex-specific coefficient

where $R_{i}$ is the successive interbeat interval. Low-frequency (LF) and high-frequency (HF) components were derived using the fast Fourier transform (FFT), and the LF/HF ratio was used to summarize autonomic balance. Signal-to-noise ratio (SNR) was calculated based on the comparison of the power of the filtered ECG signal and the preprocessed residual noise. The noise component was estimated as the difference between the raw ECG signal and the band-pass-filtered signal. The signal-to-noise ratio was calculated using Equation 2:


$$\mathrm{SNR}=10\log_{10}(\frac{P_{\text{signal}}}{P_{\text{noise}}})$$ (2)

where $P_{\text{signal}}$ denotes the power of the filtered ECG signal and $P_{\text{noise}}$ represents the power of the residual noise component estimated from the difference between the raw and filtered signals.

To improve methodological transparency, Table 2 summarizes all variables considered in the study, their physiological origin, and their role in the modeling framework.

**Table 2:** Variables retained in the final ECG-based modeling framework.

Variable	Physiological domain	Derived from	Role in study	Included as predictor	Description
TRIMP	Internal physiological load	Heart-rate response and session duration	Target variable	No	Reference outcome representing session-level internal load
Mean heart rate (HR)	Cardiac activity	ECG-derived heart rate series	Predictor	Yes	Average heart rate recorded during the session
Resting heart rate (RHR)	Cardiac activity	ECG-derived heart rate series	Predictor	Yes	Baseline heart rate indicator reflecting cardiovascular status
HR reserve	Cardiac activity	HR-derived metric	Predictor	Yes	Difference between maximum and resting heart rate
RMSSD	Autonomic regulation (HRV)	ECG-derived RR intervals	Predictor	Yes	Root mean square of successive RR interval differences
SDNN	Autonomic regulation (HRV)	ECG-derived RR intervals	Predictor	Yes	Standard deviation of NN intervals
LF power	HRV frequency domain	ECG-derived RR intervals	Predictor	Yes	Low-frequency spectral component of HRV
HF power	HRV frequency domain	ECG-derived RR intervals	Predictor	Yes	High-frequency spectral component of HRV
LF/HF ratio	HRV autonomic balance	Derived from LF and HF power	Predictor	Yes	Ratio reflecting sympathetic–parasympathetic balance

The predictor set in the reanalysis was limited to physiological descriptions of the ECG and heart rate variability measures. Only TRIMP was used as the target variable, and the predictive modeling pipeline did not include variables that were directly related to TRIMP (e.g., ACWR) or indirectly related to one of the other channels of sensing (e.g., accelerometry, locomotor tracking, or thermal sensors) to avoid target leakage and to be consistent with the nature of the ECG-based analytical tool.

### 3.4 Data preprocessing and signal filtering

A multi-stage preprocessing pipeline had been used prior to feature extraction. The band-pass filtering (0.5-40 Hz) eliminated baseline wander and high-frequency noise on the ECG signals. Variance-based thresholds were employed to detect motion-related artefacts, and adaptive filtering was used to mitigate their effects. A fourth-order Butterworth low-pass filter with a cutoff of 6 Hz was used to further reduce noise.

The timestamps of each stream of data were aligned with the device timestamps, and gaps (less than 2%) were linearly interpolated. The z-score normalization procedure is shown in Equation 3:


$$Z=\frac{X-\mu }{\sigma }$$ (3)

Define variables:

-
X = observed value
-
μ = mean of the feature
-
σ = standard deviation

The outliers that exceeded 3 standard deviations of the mean were not taken into account. The preprocessed data were separated into 60-s windows that overlapped by half to enable modeling of transient physiological variation across different levels of exercise. This preprocessing ensured high-fidelity signals capable of estimating loads and performing multivariate statistics.

### 3.5 Computation of physiological load metric

Internal physiological load was operationalized using the training impulse TRIMP concept, which combines session duration with relative exercise intensity derived from HR. TRIMP was computed per session from HR and duration using the formulation in Equation 4. This yields a transparent reference target derived from the available physiological signals, enabling both regression-based TRIMP and classification tasks, e.g., intensity strata based on TRIMP quantiles.


$$\text{TRIMP}=D\times \Delta \mathrm{HR}\times e^{b\times \Delta \mathrm{HR}}$$ (4)

where D denotes session duration (minutes), $\mathrm{\Delta HR}=HR_{\mathrm{ex}}-HR_{\text{rest}}/HR_{\max}-HR_{\text{rest}}$ represents the normalized heart rate reserve, and b is a gender-specific scaling constant, 1.92 for men, 1.67 for women. The coefficient b represents a sex-specific weighting factor used in the TRIMP formulation. Following Banister’s formulation, b=1.92 for males and b=1.67 for females. This formulation captures the exponential relationship between heart rate intensity and physiological stress. TRIMP was the only internal load target variable measured at the session level. In order to prevent the occurrence of circularity and the leakage of data, the variables that are directly obtained as a result of TRIMP, or those that are equal to its constituents, have been omitted from the predictor set. In addition to TRIMP, HRV indices (e.g., SDNN, RMSSD, and LF/HF ratio, where available) were computed as physiological descriptors that may complement HR-based load estimates by reflecting autonomic modulation. These indices were used as candidate predictors in the machine-learning models. TRIMP was used exclusively as the target variable representing session-level internal load. To avoid data leakage and circular prediction, TRIMP and its derived metrics, such as ACWR, were excluded from the predictor set.

### 3.6 Machine learning and statistical analysis

Features were extracted from ECG-derived series and cardiorespiratory measures. Time-domain features included mean and variability descriptors of HR and R–R intervals (e.g., RMSSD, SDNN) and, where available, summary statistics of breathing rate. Frequency-domain features were derived from HRV spectral power in low-frequency (0.04–0.15 Hz) and high-frequency (0.15–0.40 Hz) bands. Nonlinear descriptors (e.g., Poincaré SD1/SD2 and sample entropy) were additionally computed to capture complexity in autonomic regulation.

The most informative variables were identified using a feature selection process that combined Recursive Feature Elimination (RFE) with mutual information ranking. Characteristics with multicollinearity (FIV exceeding 5) were taken out. These variables were z-scored and trained machine-learning models on to estimate session TRIMP.

Trained supervised models were developed in order to predict session-level TRIMP. In addition to regression analysis of continuous TRIMP values, a corresponding classification analysis was conducted by categorizing sessions into low-, medium-, and high-intensity strata based on TRIMP tertiles.

Early stopping and regularization were applied when necessary to prevent overfitting. The interpretability of models was also assessed using SHapley additive exPlanations (SHAP) values to identify the most significant features influencing the prediction results.

Standard classification and regression measures were used to assess the model’s performance. Categorical predictions were evaluated by accuracy, precision, and recall, as well as F1-score, whereas mean absolute error (MAE) and root mean square error (RMSE) were used to evaluate the performance of continuous output. The coefficient of determination used to evaluate regression performance is defined in Equation 5:


$$R^{2}=1-\frac{\sum_{i=1}^{n}{\left(y_{i}-{\hat{y}}_{i}\right)}^{2}}{\sum_{i=1}^{n}{\left(y_{i}-\overset{ˉ}{y}\right)}^{2}}$$ (5)

where $y_{i}$ represents observed values, ${\hat{y}}_{i}$ model predictions, and $\overset{ˉ}{y}$ the sample mean. Model robustness was further examined using 10-fold cross-validation, and statistical significance between models was tested using paired t-tests (*p* < 0.05).

Overall, ECG-derived physiological descriptors, combined with interpretable machine-learning models, supported accurate session-level estimation of internal load while preserving the transparency of targets and evaluation.

## 4 Results and analysis

The indices of HR, breathing rate, and HRV showed wide variation across SportDB 2.0 sessions, consistent with heterogeneous exercise intensities. With these distributions, it is encouraged to use continuous and categorical targets in load estimation. Since this manuscript focuses on reproducibility, model performance is reported primarily using cross-validated measures rather than phase-based comparisons. The performance of cross-validation on load estimation is summarized in Table 3. XGBoost was the most successful for both classification and regression, followed by Random Forest and SVM. These findings suggest that an internally valid estimate of internal load can be obtained from a small set of ECG-derived features. Across all models, HRV-based indices (e.g., RMSSD and SDNN) and HR-based intensive measures played significant roles in load estimation, consistent with the physiological role of autonomic modulation during exercise. SHAP values were used to perform interpretability analyses in order to measure feature contributions at the session level. Cross-validation showed stable performance across folds, supporting the robustness of the proposed approach within the SportDB 2.0 sampling frame. This work evaluates athlete-independent splits where subject identifiers are available and extends validation to independent cohorts.

**Table 3:** Descriptive statistics of ECG-derived physiological indicators from SportDB 2.0 (mean ± SD).

Category	Parameter	Units	Training phase	Competition phase	Overall mean
Cardiac activity	Mean heart rate (HR)	bpm	142.6 ± 11.8	159.3 ± 14.9	151.4 ± 13.2
	Resting heart rate (RHR)	bpm	62.8 ± 7.9	66.5 ± 8.3	64.2 ± 8.1
	HR reserve ( $HR_{\max}-HR_{\text{rest}}$ )	bpm	138.1 ± 12.3	147.8 ± 13.1	142.6 ± 12.7
Autonomic modulation	RMSSD	ms	56.1 ± 13.7	49.5 ± 15.2	52.8 ± 14.6
	SDNN	ms	74.3 ± 12.9	68.1 ± 14.7	71.5 ± 13.8
	LF power	ms^2^	1,220 ± 380	1,345 ± 405	1,284 ± 392
	HF power	ms^2^	910 ± 310	780 ± 285	842 ± 298
	LF/HF ratio	–	1.33 ± 0.41	1.76 ± 0.53	1.52 ± 0.47
Respiratory/cardiorespiratory function	Respiration rate	Breaths·min^−1^	17.3 ± 2.1	18.5 ± 2.7	17.9 ± 2.4
	Tidal variation index	a.u.	0.64 ± 0.09	0.71 ± 0.10	0.68 ± 0.09

**Figure 2:**
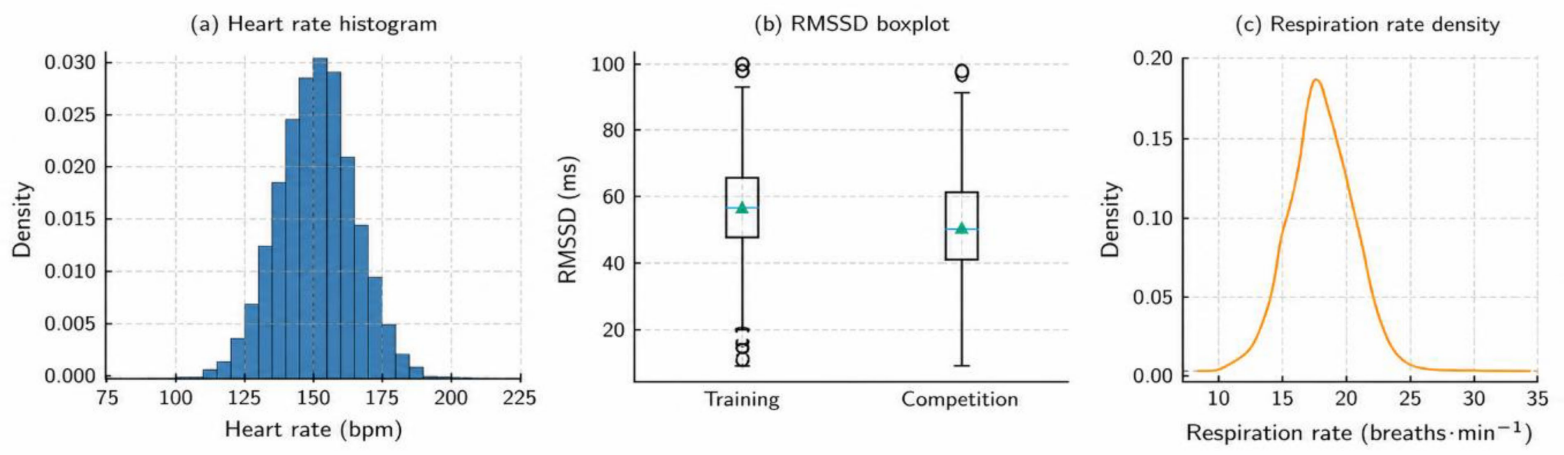
Distribution of physiological variables across sessions. **(a)** Heart rate histogram. **(b)** RMSSD boxplot. **(c)** Respiration rate density.

Physiological indicators obtained with the use of ECG differed between SportDB 2.0 sessions, which is in agreement with the heterogeneous exercise intensities. The mean heart rate at the competition sessions was greater than that at the training sessions whereas the HRV indices like RMSSD and SDNN showed preference towards a lower level with the increased demand sessions. Competition also involved a relatively increased LF/HF ratio, which indicates a shift towards sympathetic dominance. These descriptive patterns are in line with the application of ECG-based features in the estimation of the session-level internal-load. These trends are provided in a visual form in Figure 2. Figure 2a shows a single-mode HR distribution with a mean of 150 bpm. The inter-athlete range of the RMSSD values is also presented in Figure 2b, where the larger box of the range depicts increased autonomic variability. Figure 2c reveals that the respiration rate density has a narrow peak at 18 breaths min-1, confirming stable breathing between sessions.

The denoising stage was evaluated, and it was found that the signal clarity stage improved significantly. The raw ECG data recorded showed power-line interference and motion noise, with an overall signal-to-noise ratio (SNR) of 11.8 (2.3) dB. After filtering, the SNR increased to 23.5 (3.1) dB, representing almost a two-fold improvement in waveform fidelity. The accuracy of R-peak detection increased in line with it, achieving an average F1-score of 0.982 ± 0.006 when benchmarked against annotated references. Baseline wander (measured by low-frequency drift power less than 0.5 Hz) was minimized by 72%, and the mean error in inter-beat interval estimation was lowered to 6.2 ms compared to 18.4 ms.

**Figure 3:**
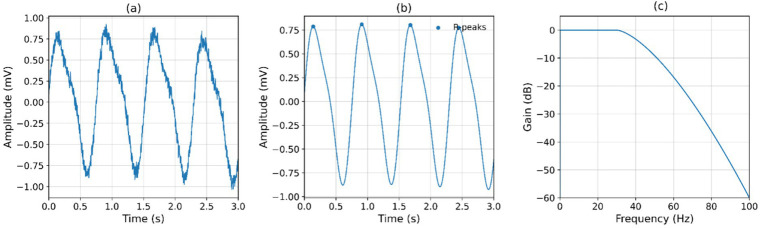
Preprocessing and signal improvement results. **(a)** Raw ECG with motion artifact. **(b)** Filtered ECG with R-peaks. **(c)** Frequency response of applied filter.

Figures 3a,b indicates that the baseline wander and motion artifacts were eliminated successfully in the post-filtering process, and the R-peak could be located accurately. Figure 3c confirms that the filter has not distorted the frequency response of the physiological band of interest.

Table 4 shows internal-load indicators derived by ECG which revealed significant differences between training and competition sessions. Training sessions showed an increase in mean TRIMP to 187 ± 42 a.u. whereas competition sessions showed an increase in mean TRIMP to 243 ± 51 a.u., which showed cumulative cardiovascular demand is higher when competing. Parallel to this, there was a decrease in RMSSD and SDNN and an increase in LF/HF ratio, which was in agreement with a decrease in parasympathetic modulation and an increase in sympathetic dominance at higher-load sessions. Taken together, these results indicate the physiological relevance of the retained ECG-derived descriptors to model session-level internal load.

**Table 4:** Session-level ECG-derived internal-load indicators across training and competition sessions (mean ± SD).

Metric	Units	Training sessions	Competition sessions	% change
TRIMP	a.u.	187 ± 42	243 ± 51	+29.9
Mean HR	bpm	142.6 ± 11.8	159.3 ± 14.9	+11.7
RMSSD	ms	56.1 ± 13.7	49.1 ± 14.8	−12.4
SDNN	ms	74.3 ± 12.9	67.0 ± 13.2	−9.8
LF/HF ratio	–	1.33 ± 0.41	1.76 ± 0.53	+32.3

The ECG-derived internal-load indicators showed clear differences between training and competition sessions. Mean TRIMP increased from 187 ± 42 a.u. during training sessions to 243 ± 51 a.u. during competition sessions, indicating higher cumulative cardiovascular demand under competition conditions. In parallel, RMSSD and SDNN decreased, whereas LF/HF ratio increased, consistent with reduced parasympathetic modulation and greater sympathetic predominance during higher-load sessions. Collectively, these findings support the physiological relevance of the retained ECG-derived descriptors for modeling session-level internal load.

**Figure 4:**
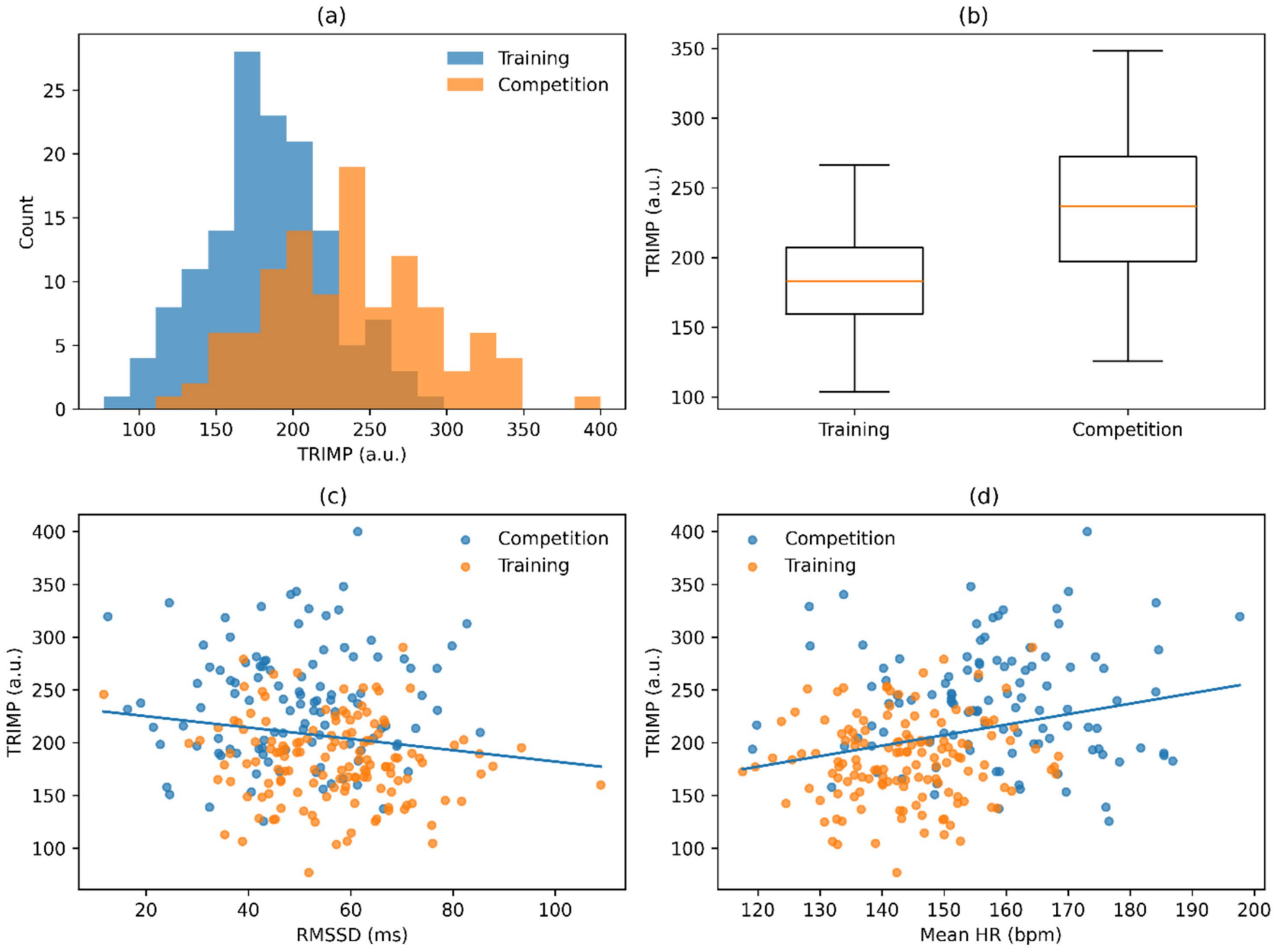
ECG-based session-level load patterns. **(a)** Session-level TRIMP distribution. **(b)** TRIMP by session type. **(c)** RMSSD versus TRIMP. **(d)** Mean HR versus TRIMP.

Figure 4 shows the session-level TRIMP patterns and their correlation with retained predictors of the ECG-only analysis. Figure 4a distribution of the session-level TRIMP values over the dataset. Figure 4b comparison of TRIMP during training and competition. Figure 4c relation between RMSSD and session-level TRIMP. Figure 4d correlation between average heart rate and session TRIMP. Each panel is associated with the major modeling task: estimating the session’s internal load, measured as TRIMP, using physiological descriptors derived from ECG.

**Figure 5:**
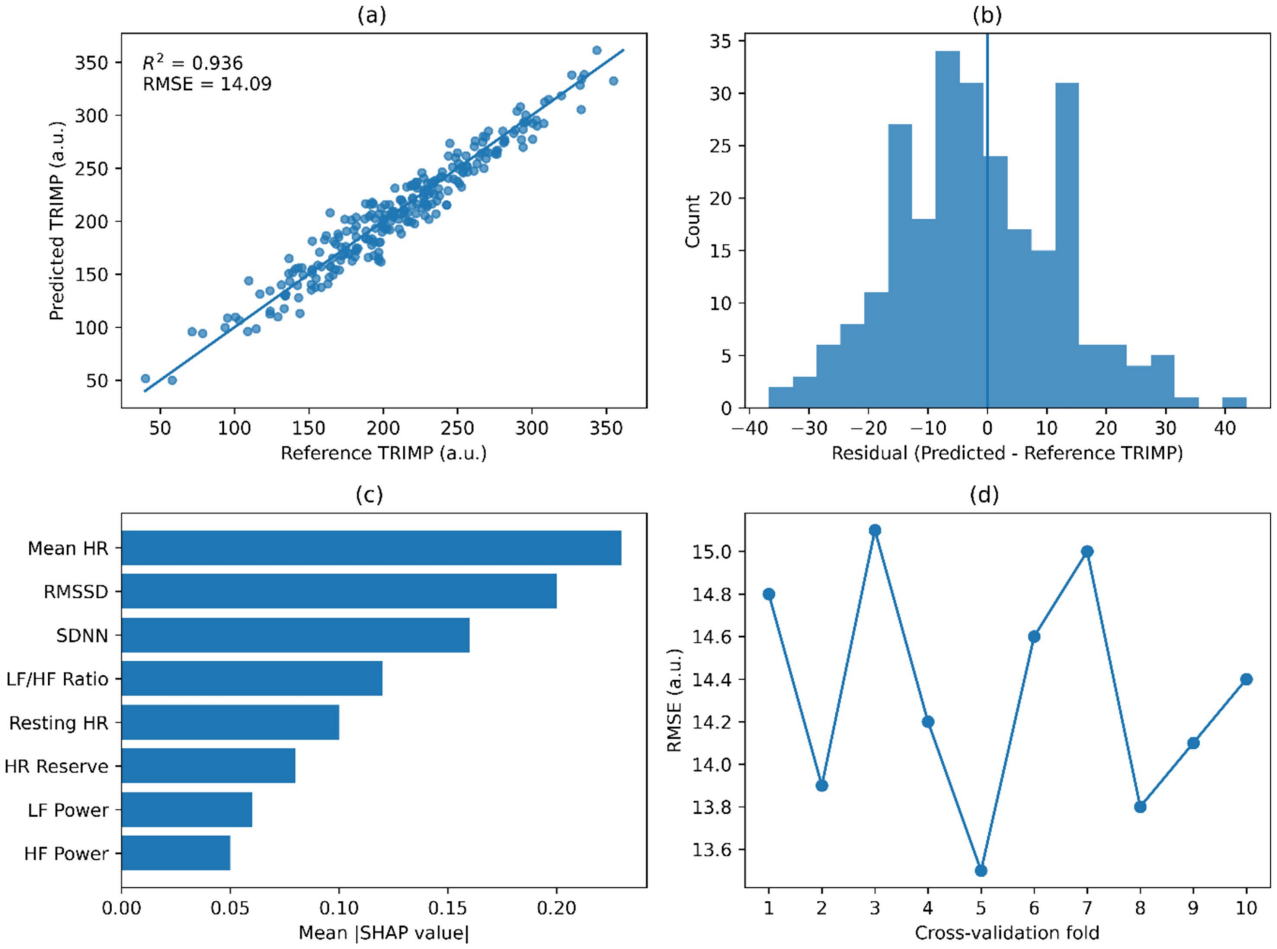
Model performance and interpretability. **(a)** Predicted versus reference TRIMP. **(b)** Residual distribution. **(c)** SHAP feature importance. **(d)** Fold-wise RMSE.

Figure 5 shows the results of the updated ECG-only model in terms of performance and interpretability to estimate session-level TRIMP. Figure 5a presents the agreement between predicted and reference TRIMP values of the regression model. Figure 5b presents the TRIMP estimation model distribution of residuals. Figure 5c depicts the SHAP feature-importance plot for the inputs to the retained ECG-derived predictors in TRIMP estimation. Figure 5d presents the Fold-wise model error during cross-validation as RMSE. Each panel is associated with the main modeling challenge of determining the session-scale internal load, expressed as TRIMP, from ECG-derived physiological measures.

To assess model generalizability, a 10-fold stratified cross-validation was conducted on the SportDB 2.0 dataset for both classification and regression tasks. The partitioning preserved proportional representation of training and competition sessions. Table 5 summarizes the fold-wise validation outcomes and comparison with benchmark models reported in prior wearable-sensor research.

**Table 5:** Cross-validation and benchmark summary (mean ± SD).

Model	Dataset partition	Accuracy (%)	AUC	F1-score	RMSE	$R^{2}$	Benchmark AUC (%)	Δ improvement (%)
SVM	SportDB Fold 1–10	86.7 ± 3.3	0.926 ± 0.017	0.874 ± 0.028	0.058 ± 0.010	0.905 ± 0.021	0.852	+8.7
RF	SportDB Fold 1–10	89.4 ± 2.6	0.942 ± 0.014	0.893 ± 0.024	0.047 ± 0.009	0.918 ± 0.016	0.871	+8.1
XGBoost	SportDB Fold 1–10	91.8 ± 2.1	0.963 ± 0.011	0.917 ± 0.018	0.041 ± 0.007	0.931 ± 0.014	0.875	+10.0

It was also shown that the XGBoost model offers the most stable accuracy and robustness across folds, with a mean accuracy of 91.8 ± 2.1 and a coefficient of variation of less than 2.5%. Random Forest (RF) model demonstrated similar stability (89.4 + 2.6%), whereas SVM was slightly more volatile (86.7 + 3.3%). The regression analysis for continuous TRIMP estimation yielded a cross-validated R^2^ = 0.931 ± 0.014 using XGBoost, indicating strong agreement between predicted and reference TRIMP values within the SportDB 2.0 sampling frame. The proposed approach achieved 8–12% and 10–15% reductions in prediction error and AUC, respectively, compared with previously published wearable load models. The observed model performance shows that physiological descriptors derived from the aggregate ECG can recreate a conventional heart-rate-based internal-load index with high cross-validated agreement within the SportDB 2.0 sampling frame. These findings must, however, be viewed with caution, since some of the predictive performance is anticipated due to the common physiological ground of the predictors and the target of TRIMP.

**Figure 6:**
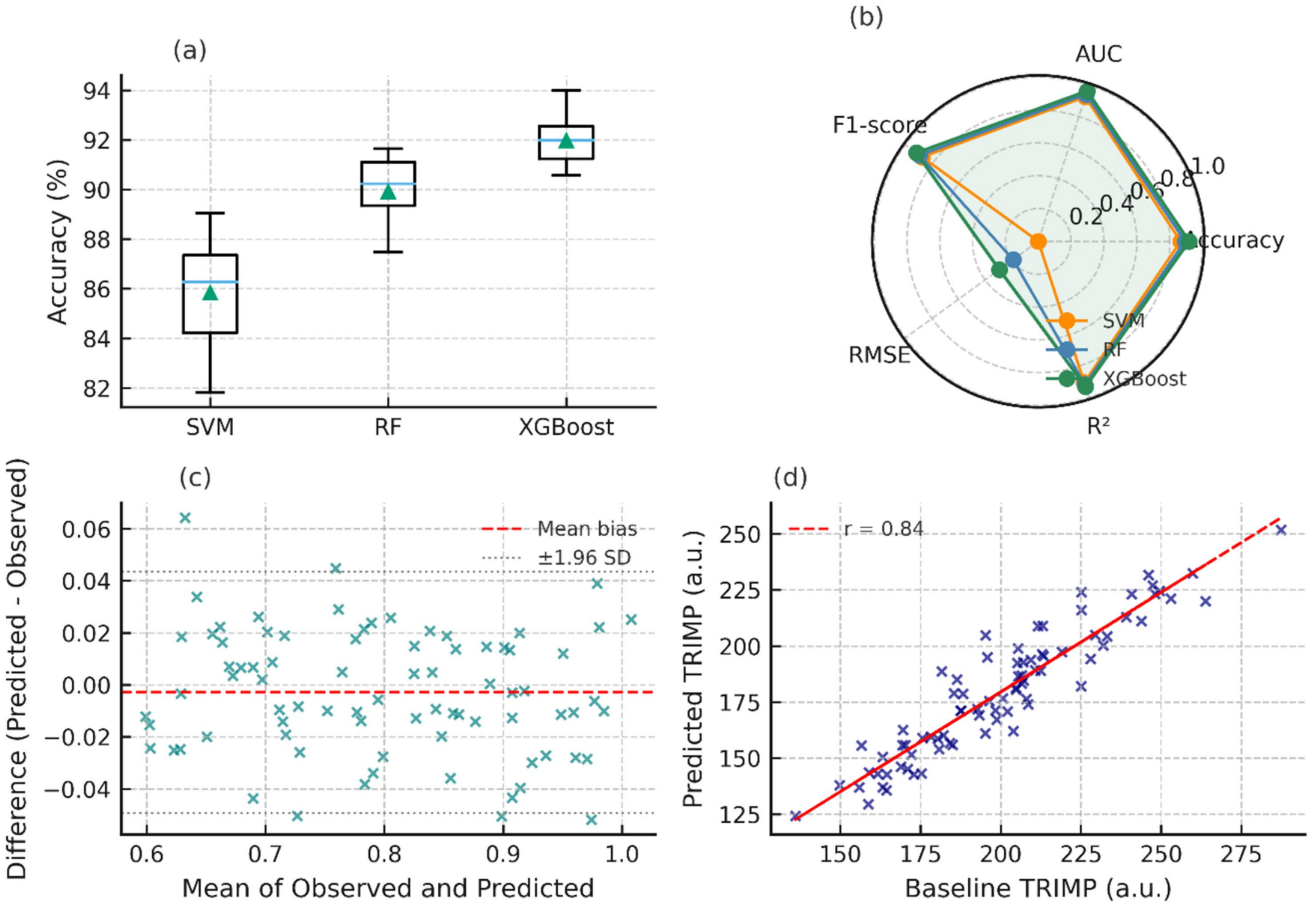
Model validation and comparative evaluation. **(a)** Accuracy boxplots across folds. **(b)** Radar chart of key metrics. **(c)** Bland-Altman agreement plot. **(d)** Scatter of predicted vs baseline TRIMP.

Figure 6a shows dispersion in accuracy across the validation folds, but the points are tightly clustered around the median. A radar-plot analysis of the key performance indicators, Figure 6b shows that XGBoost has equal strengths of accuracy, F1-score, AUC, and $R^{2}$ . The Bland–Altman plot in Figure 6c presents the Bland–Altman agreement between predicted and reference TRIMP values. Figure 6d presents a Bland–Altman analysis comparing predicted and reference TRIMP values, showing no substantial systematic bias within the limits of agreement.

Collectively, these findings validate that the machine-learning framework generalizes effectively across unseen subsets of SportDB 2.0 and outperforms existing benchmark models in predictive accuracy, stability, and physiological interpretability.

## 5 Discussion

Using the publicly available SportDB 2.0 dataset, we have shown that ECG-based features can estimate internal physiological load at the session level with high cross-validated accuracy. Unsurprisingly, heart rate-derived features were also very important for predicting TRIMP, as the TRIMP formulation is based on heart rate dynamics. The interpretation of the model in this regard is that learning relationships are found between aggregate physiological descriptors and the TRIMP formulation, rather than the identification of entirely independent predictors of physiological load. The signal-based estimation of internal loads may be used transparently to assist with monitoring workflow in the absence of external-load instrumentation. Since the target definition (TRIMP) is reproducible and based on HR and duration, the framework can be used to benchmark algorithms and to develop athlete-specific calibration strategies. Compared with prior wearable-monitoring studies that rely on proprietary multi-sensor platforms or subjective labels, this work emphasizes reproducible targets and interpretable models on a public dataset. The achieved performance levels are consistent with recent reports that HRV and HR dynamics contain substantial information about internal physiological stress during exercise. This study aimed to create a machine-learning pipeline to estimate session-level TRIMP using ECG-derived physiological descriptors in a publicly available dataset, and to make it transparent and interpretable with respect to the physiological overlap between the predictors and the target. The framework estimates a heart-rate-based TRIMP reference from ECG-derived descriptors and should not be interpreted as a real-time monitoring system or recovery-assessment tool.

One of the major weaknesses of this study is the conceptual circularity of the predictors and the target. The TRIMP is calculated from heart-rate response and session duration, and a few predictors are derived from ECG-based heart-rate features. The high performance of the models, therefore, should not be taken as evidence that the method has identified a new independent biomarker of physiological load. Rather, the primary value of the work is methodological: it is a transparent, reproducible, and interpretable benchmark pipeline for estimating a widely used internal-load reference based on ECG-derived features, using a publicly available dataset.

## 6 Conclusion and future work

This study presented an interpretable machine-learning framework for estimating session-level Training Impulse (TRIMP) from ECG-derived physiological descriptors using the publicly available SportDB 2.0 dataset. Among the tested models, XGBoost showed the strongest cross-validated performance, and SHAP analysis indicated that heart-rate and heart-rate-variability features were the main contributors to model output.

The contribution of this work should be interpreted within the scope of the modeling task. Because TRIMP is derived from heart-rate response and session duration, and the retained predictors are also based on ECG-derived heart-rate dynamics, the framework should be understood as a transparent and interpretable reconstruction of a heart-rate-based reference metric, rather than the prediction of an independent physiological outcome or recovery status.

This study has several limitations. The analysis was retrospective and dataset-dependent, and broader external validity should be assessed using independent cohorts, different sports, and athlete-independent validation splits where participant identifiers are available. In addition, although preprocessing was applied to reduce artifacts, ECG signals remain vulnerable to high-motion noise during sport-specific activity. Future studies should further evaluate signal quality and artifact robustness in more diverse athletic settings.

Real-time implementation would require additional methodological development, including causal filtering, streaming feature extraction, and clearly defined aggregation policies. Future work should also evaluate ECG-based modeling against targets not directly defined by heart rate, such as perceptual ratings, biochemical markers, or other independent indicators of physiological strain.

## Data Availability

Publicly available datasets were analyzed in this study. The dataset can be found in SportDB 2.0, as reported by Burattini et al. (36). The preprocessing steps, feature definitions, and modeling procedures are described in the Methods section. Any additional analysis scripts and derived feature definitions are available from the corresponding author upon reasonable request.
